# Model based dynamics analysis in live cell microtubule images

**DOI:** 10.1186/1471-2121-8-S1-S4

**Published:** 2007-07-10

**Authors:** Alphan Altınok, Erkan Kiris, Austin J Peck, Stuart C Feinstein, Leslie Wilson, BS Manjunath, Kenneth Rose

**Affiliations:** 1 Department of Electrical and Computer Engineering, University of California – Santa Barbara, Santa Barbara, CA 93106, USA; 2 Department of Molecular, Cellular, and Developmental Biology, University of California – Santa Barbara, Santa Barbara, CA 93106, USA

## Abstract

**Background:**

The dynamic growing and shortening behaviors of microtubules are central to the fundamental roles played by microtubules in essentially all eukaryotic cells. Traditionally, microtubule behavior is quantified by manually tracking individual microtubules in time-lapse images under various experimental conditions. Manual analysis is laborious, approximate, and often offers limited analytical capability in extracting potentially valuable information from the data.

**Results:**

In this work, we present computer vision and machine-learning based methods for extracting novel dynamics information from time-lapse images. Using actual microtubule data, we estimate statistical models of microtubule behavior that are highly effective in identifying common and distinct characteristics of microtubule dynamic behavior.

**Conclusion:**

Computational methods provide powerful analytical capabilities in addition to traditional analysis methods for studying microtubule dynamic behavior. Novel capabilities, such as building and querying microtubule image databases, are introduced to quantify and analyze microtubule dynamic behavior.

## Background

Microtubules (MTs) are filamentous cytoskeletal structures composed of tubulin protein subunits. These subunits can associate with, or dissociate from, existing tubulin polymers rapidly, making MTs highly dynamic. Through these dynamic behaviors, MTs are critically involved in many essential cellular functions. MT dynamics are finely regulated in the cell, [1]. It has been hypothesized that inadequate regulation of neuronal MT dynamics may underlie neuronal cell death in Alzheimer's and related dementias, [2]. Additionally, drug induced modulation of MT dynamics underlies the effectiveness of various anti-cancer drugs, such as Taxol, [3]. For these and a host of basic biology issues, the regulation of MT dynamics is a very active area of research in modern cell biology.

**Figure 1:**
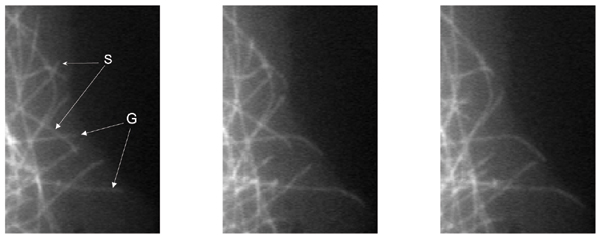
Consecutive time-lapse images of MTs taken at 4 sec. intervals. Examples of growing (G) and shortening (S) MTs are marked. Tip locations of these MTs are manually tracked over time by marking on consecutive frames to calculate the growth and shortening statistics.

**Figure 2:**
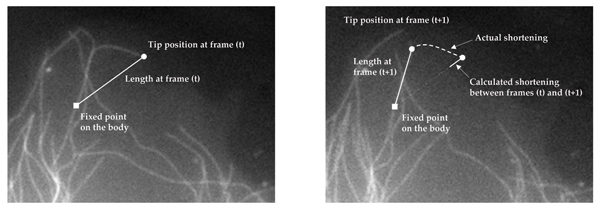
In each frame, length of a MT is estimated by the Euclidean distance between a fixed point on the MT, called *the origin*, and the MT tip, (a). Shortening length between two consecutive frames is calculated as the difference of respective lengths. This estimate may not reflect the actual shortening as shown in (b).

A key tool of MT dynamics research is to track the growing and shortening behaviors of individual MT tips from time-lapse images (Fig. 1), and quantitatively describe MT behavior under different experimental conditions. Traditional MT dynamics parameters consist of statistics derived from the *growth *and *shortening *events between consecutive frames. In general, tracking is a largely manual and laborious task, [4]. Furthermore, it is approximate (Fig. 2), variable between users and labs, and potentially biased for more dynamic MTs, [5]. The resulting quantification and analysis capabilities are limited with manual feasibility. For example, while MT deformations may contain valuable information in studying neuronal growth-cone path finding, it is impractical to manually collect relevant data, e.g. curvature or orientation, from many MTs. Additionally, due to the laborious nature of manual data collection, a limited sample for each experimental condition must represent all MTs collected in that condition. While different subsets of MTs undertake distinct tasks in the cell, and therefore can exhibit distinct dynamic characteristics, generally there are limited means of observing such dynamics in isolation through manual methods. Analysis of dynamic behavior is further limited by pairwise comparisons of behavioral features between control and treated conditions. Therefore, computational methods could make an immediate contribution to MT dynamics research.

In this work, we propose a powerful approach for analyzing MT dynamic behavior. Briefly, we use an automated tracking method for measuring MT dynamics, which are then modeled as *MT behavior patterns *by Hidden Markov Models. The proposed methods go beyond the traditional analysis capabilities and offer new insights in investigating MT dynamic behavior.

### Microtubule structure and function

The cytoskeleton of a eukaryotic cell consists of a network of fibers. MTs are one of the three principal types of cytoskeletal fibers. They are hollow cylindrical structures, 25 nm in diameter and up to several *μ*m in length, consisting of non-covalently bound tubulin protein subunits. MTs are constantly assembled and disassembled, making the cytoskeleton a dynamic system. MTs are critically involved in a number of essential cellular functions, such as chromosome segregation at mitosis and intracellular cargo transport. Additional background information on MT structure and function can be found in [1].

The growing and shortening dynamics of MTs are finely regulated, for example, by the action of *MT-associated proteins *(MAP) and *MT-targeted drugs *(MTD). A large body of evidence, reviewed by Feinstein and Wilson [2], suggest that cell viability requires that MT dynamics be properly regulated within a narrow range. Common conjecture is that certain diseases such as Alzheimers and cancer are at least correlated with the regulatory abnormalities in MT dynamics, [6-8]. Consequently, gaining a detailed mechanistic understanding of the regulatory activities of MAPs, [5,6,9], and MTDs, [3,10], is a major focus of current research. A major challenge is assessing the activities of the large number of MAPs and their many isoforms, as well as the large number of MTDs and their many derivatives. For instance, the MAP tau consists of 441 amino acids, more than 25 of which can be phosphorylated in various combinatorial patterns. Whereas phosphorylation normally serves to regulate tau activity, excessive and abnormal phosphorylation correlates with cell death and dementia. Thus, to fully understand normal and pathological tau action, the regulatory effects of the many different combinational phosphorylation patterns of tau must be understood.

### Current analysis method

MTs are polar structures, possessing biochemically distinct *minus *and *plus *ends. Conventionally, *the minus end *of a MT is assumed to be fixed at the MT organizing center near the nucleus, and the other end -*the plus end *or *the tip*- is the dynamic end that is observed in most MT dynamics studies. Typically, in live cell studies, minus ends of the MTs are not visible because of the high density of MTs converging on the organizing center. Thus, in calculating the MT length, a point on the MT body is selected as a reference point, *origin*, after an initial observation of all frames in the time-lapse images, (Fig. 2).

Traditionally, time-lapse images of MT populations are collected following treatment with MTDs or MAPs. Dynamics parameters are then manually calculated from image sequences as follows. The positions of MT plus ends are manually tracked individually across all frames, (Fig. 1). MT lengths are approximated as the (Euclidean) distance between tracked tip positions and the origin, producing MT *life histories *or *tracks*, (Fig. 2). The change in MT length is computed between consecutive frames, and growth and shortening statistics are tabulated. Length changes below a threshold are marked as *attenuation *or *pause*, signifying undetectable change. Other biologically significant events are the conversion of a MT from a growing state to rapid disassembly, designated as a *catastrophe*, and a subsequent potential recovery from shortening to attenuation or growth, called *rescue*. To estimate the effects of a regulatory agent upon MT dynamics, these statistics are aggregated over a number of MTs from the same experimental condition. Resulting statistics of each condition are compared with the control behavior to quantify the effects of the examined agent on dynamics parameters.

In this fashion, regulatory effects of each individual agent are studied through a laborious set of tasks. Quantifying sufficient image data to achieve statistical significance and limited comparative capabilities in the presence of innumerous possible agents pose an enormous challenge to researchers. Example studies are [3,5,9-12].

Statistics obtained from the growth and shortening events treat these events independently, rather than as being part of a behavior pattern. For instance, a certain growth measurement is counted as the same event regardless of where it occurs in relation to preceding or subsequent events. Furthermore, studying event correlations between neighboring MTs are generally infeasible, despite potential biological significance.

There are no established non-manual methods for examining the similarities and differences in particular dynamic behaviors imposed by various agents. Furthermore, studying combined effects of multiple regulatory agents is difficult, due to the limitations imposed by the pairwise comparisons between experimental conditions. For example, consider a hypothetical MTD *AB*, derived from MTDs *A *and *B*. In order to understand the contributions of *A *and *B*, multiple individual experiments must be conducted. Therefore, quantifying behavioral similarities across experimental conditions may provide essential guidance in constructing hypotheses.

In this work, we propose an automated tracking and analysis method to address the limitations mentioned above. The tracking component provides behavioral features for subsequent analysis. We define the *MT dynamic behavior *as a sequence of changes in MT length over equal time intervals. Experimental conditions may exhibit a number of behavioral patterns, which are estimated in parametric form by a mixture of Hidden Markov Models. By using a model-based clustering technique, we propose to analyze the constituent parts of MT behavior in each experimental condition. Thus, each experimental condition can be described as a mixture of behaviors exhibited by different MT populations. Through estimated average behavior patterns, we introduce a probabilistic behavioral distance measure between experimental conditions. Furthermore, parameters of individual models may present significant information about the properties of corresponding behavioral patterns. We describe how model-based analysis can be effective in addressing the above limitations (see Discussion).

## Results

We present statistical models of MT behavior that are estimated using automatically tracked MT dynamics data. As a comparison, we provide models of manually collected MT tracks. We describe the results of automated tracking using visual samples and associated errors.

### Quantifying microtubule dynamics by automated tracking

**Figure 3:**
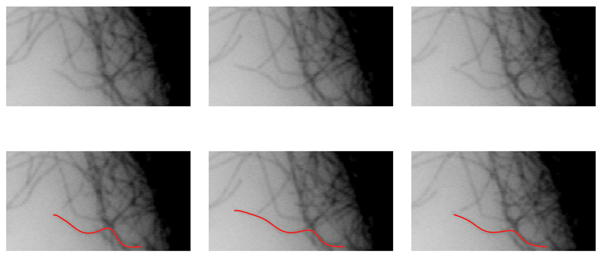
Example tracking results. Original frames are shown in (a – c). Computed MT bodies in corresponding frames are superimposed in (d – f). While the MT body trace was swayed by an intersecting MT, consistent estimation of the body trace is sufficient for quantifying the growth or shortening at the MT tip.

**Figure 4:**
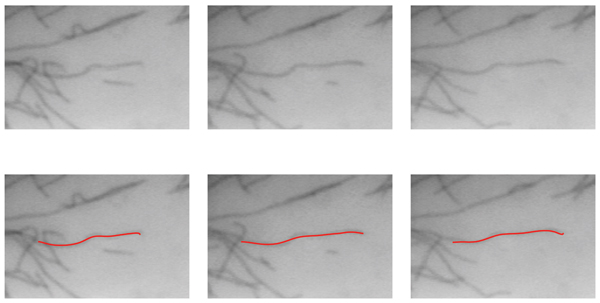
Example tracking results. Original frames are shown in (a – c). Computed MT bodies in corresponding frames are superimposed in (d – f).

**Figure 5:**
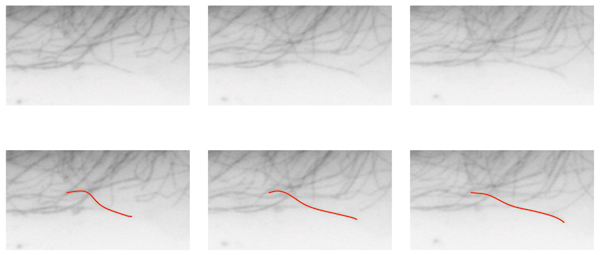
Example tracking results. Original frames are shown in (a – c). Computed MT bodies in corresponding frames are superimposed in (d – f). This example displays the small variations on the estimated *origin*. As a consequence of the *minus end *estimation procedure, this variation is the main component of the errors in length computation.

**Figure 6:**
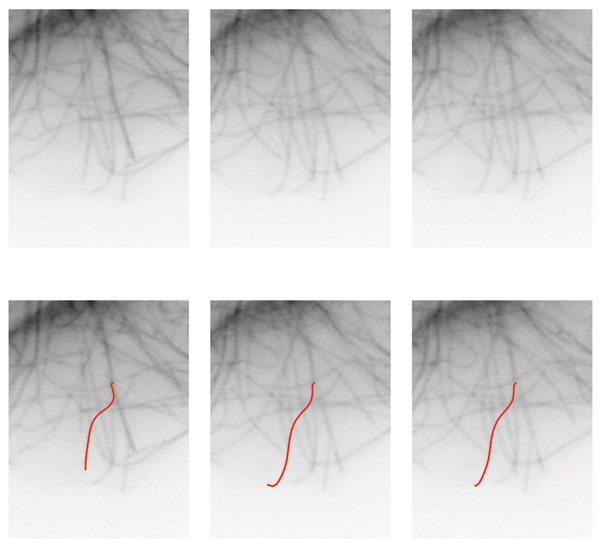
Example tracking results. Original frames are shown in (a – c). Computed MT bodies in corresponding frames are superimposed in (d – f).

**Figure 7:**
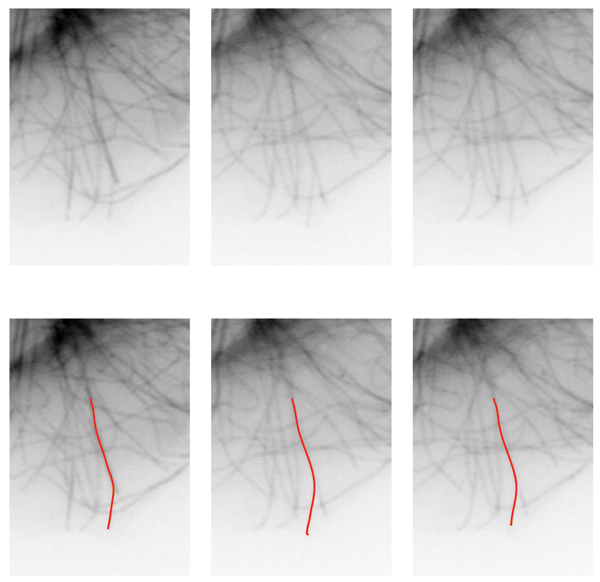
Example tracking results. Original frames are shown in (a – c). Computed MT bodies in corresponding frames are superimposed in (d – f).

For quantifying MT growth and shortening, we used the tracking method proposed in [13]. In the spatiotemporal graph matching (see Methods), up to three missing frames between tips of the same MT track were allowed. The computation of the geodesics, the distances for the weights on the graph, and the selection of a fixed point on the MT body were carried out using the Fast Marching algorithm, [14]. Visual tracking results are shown in (Fig. 3, 4, 5, 6, 7).

Quantitative results of MT tracking were given in [13]. Evaluations against manually tracked data shows that the mean and the standard deviation of tracking error are 2.85 and 4.36 pixels, respectively. This error level is acceptable for biological studies. Recall that the MT width is 25 nm (see Background), which appears as curves that are 3 pixels in width. Thus, a growth or shortening event that is less than 3 pixels would correspond to an event that is too small to quantify reliably, and is considered as *attenuation*.

We note that the tracking performance is sensitive to the accuracy of initial tip detection step. Furthermore, the proposed approach requires multiple tips to be detected for reliable extraction of MT tracks by design. In other words, the tracking performance may be adversely affected in tracking MTs individually, which may limit the ability to track a particular MT in a cell. Finally, intersecting MTs may *steal *the body trace, as the geodesic distance will favor higher intensity levels, (Fig. 3d – 3f). This issue could be addressed with further constraints on the MT orientation and curvature. However, in this work, we limit the behavioral features to the observed change of length in the MT plus end, which only requires consistent estimation of the MT body.

While tracking performance may be improved as a consequence of higher image quality and suitable algorithms targeting frequent intersections, deformations, and intensity variations. In its current state, automated tracking can track and quantify 10 times more MT tracks per image sequence than manual methods. With this increase in analyzable data volume, we are able to estimate behavior models for different experimental conditions. Estimated statistical models of MT dynamic behaviors are presented in the next section.

### Statistical models of microtubule behavior

In this work, we used MT time-lapse live cell images from [10]. The authors of [10] investigate the hypothesis that resistance to Taxol may involve altered sensitivity to different tubulin isotypes. Chinese hamster ovary (CHO) cells were microinjected with rhodamine-labeled tubulin. A total of 111 sequences were acquired using fluorescence microscopy with a 100× objective lens (1000× magnification). 25 frames per sequence were captured at 4 second intervals, from five different conditions.

Growth and shortening rates were computed as the differences of a MT lengths between consecutive frames, measured in pixels. Thus, each track consists of an observation sequence composed of 25 points in time. Resulting observation sequences were in the range [-13.03, 11.22] pixels, where (-) and (+) denoting shortening and growth rates, respectively.

The study in [10] analyzes the potential for Taxol (a cancer therapeutic) resistance in cells expressing different tubulin isoforms. Five experimental conditions were recorded, Table 1. Results in [10] show that two groups of *EC *exhibit different dynamics: {*EC*_1_, *EC*_2_, *EC*_4_} vs. {*EC*_3_, *EC*_5_}, where the MTs in the first group are more dynamic than the ones in the second group. It is also reported that *EC*_4 _is more dynamic than *EC*_5_. In this work, we evaluated our modeling approach using both automatically (3068 tracks) and manually (210 tracks) tracked MTs, Table 1.

**Table 1:** Experimental conditions and number of tracks collected, automatically (AT) and manually (MT).

	Experimental condition	AT	MT
*EC*_1_	*β*III-tubulin expressed, no Taxol	897	58
*EC*_2_	*β*III-tubulin expressed, plus Taxol	614	33
*EC*_3_	*β*III-tubulin not expressed, plus Taxol	414	17
*EC*_4_	*β*I-tubulin expressed, no Taxol	370	30
*EC*_5_	*β*I-tubulin expressed, plus Taxol	773	72

The first experiment was designed to confirm biological results. A classification score between *EC*_4 _and *EC*_5_, denoted by EX:A, and between condition groups {*EC*_3_, *EC*_5_} and {*EC*_1_, *EC*_2_, *EC*_4_}, denoted by EX:B, were computed with a 3-way cross-validation, Table 2. Well defined separations between the two groups and between Taxol-treated and control tracks agree with established biological findings. A third test, denoted by EX:C, was aimed to separate *EC*_3 _from *EC*_5_. Biological results indicate that these experimental conditions exhibit highly similar dynamics. A maximum separation of much less than EX:A and EX:B verify this finding.

**Table 2:** Correct classification rates for EX:A, B, C.

	EX:A	EX:B	EX:C
Correct AT (%)	95.91	94.27	62.67
Correct MT (%)	92.16	86.96	66.67

First row shows results from automatically tracked MTs, second row shows results from manually tracked MTs.

The same set of experiments were repeated with manually tracked MT data. Separation results are shown in Table 2. Similar classification rates with the automatically tracked experiments confirm the automated tracking as well as the applicability of model based analysis.

Our HMM implementation was derived from [15]. Experimentation with both left-right and fully connected HMMs revealed that fully connected models were better suited for the modeling task, in line with biological input. Growth and shortening rates were assumed to be drawn from Gaussian emissions. It should be noted that the number of larger growth and shortening events decrease exponentially as the length of the event increases. Therefore, using exponential emission distributions may be appropriate. However, detection of events measuring less than 3 pixels may be unreliable for both manual and automated tracking (see Current analysis method). Since good initialization values are essential with continuous emission distributions, we derived statistics from observation vectors for initializing emissions. Transition and state priors were initialized randomly, and the number of clusters was determined experimentally, Table 3.

**Table 3:** Change in correct classification rates vs. the number of models from EX:B. Separation peaks at *W *= 3.

*W*	1	2	3	4	5
Correct (%)	62.11	76.28	94.27	72.33	57.44

Ultimately, statistics collected by the model parameters are more significant in biological studies than the classification scores. To that end, we examine the models of each *EC*. Table 4 shows emission distributions of selected component models used in EX:A. The models were estimated by using automatically tracked MTs. Table 5 shows the corresponding models estimated with manual tracks. The first rows in each model correspond to the mean length change captured by that model state (*q*_*i*_), where negatives indicate shortening. Nearly all states of *λ*_4 _show stable distributions, while states in *λ*_5 _show significantly more dynamic behavior. Both models have states exhibiting stable growth and shortening, indicating that the main discriminating factor between the two behavior patterns are the large growth and shortening events occurring occasionally. Naturally, the average growth and shortening rates captured in model states are direct results of the observations, and they confirm that Taxol-treated MTs show suppressed dynamics with *β*I-tubulin than non-treated MTs.

**Table 4:** Example emission distributions of *λ*_1 _from *EC*_4_, and *λ*_2 _from *EC*_5_.

AT		*q*_1_	*q*_2_	*q*_3_	*q*_4_
*λ*_1_	*μ*	4.03	-2.42	0.48	0.01
	*σ*	2.17	2.59	0.91	8.08
*λ*_2_	*μ*	0.58	0.32	0.56	0.22
	*σ*	0.61	3.32	0.65	8.32

Models were trained using automatically extracted tracks.

**Table 5:** Example emission distributions of *λ*_1 _from *EC*_4_, and *λ*_2 _from *EC*_5_.

MT		*q*_1_	*q*_2_	*q*_3_	*q*_4_
*λ*_1_	*μ*	3.29	0.74	-2.38	0.01
	*σ*	4.20	0.02	2.52	0.01
*λ*_2_	*μ*	-0.35	-1.62	1.89	3.55
	*σ*	1.31	8.01	1.59	12.17

Models were trained using manually extracted tracks.

## Discussion

Estimated models can provide more descriptive information about the behavior patterns than what is available through manual methods: (*i*) typical growth and shortening states of the modified behavior, and (*ii*) the transition probabilities between these states. For example, as a direct comparison with manual methods, besides the traditional *catastrophe *and *rescue *frequencies, transitions from small to larger events of the same type can be quantified. In essence, characteristics of behavior patterns are parametrically encoded in models, which can then be used in generating these behaviors. We describe further model-based analysis capabilities in the next section.

### Novel analytical capabilities

The proposed approach provides a number of novel analytical capabilities (see Background). The most important aspect of this approach is using entire MT life histories as opposed to parsing the events into predefined categories. Therefore, events are evaluated for their contribution in different behavior patterns. With the introduction of this method, it becomes possible to compare effects of regulatory agents at different levels: (*i*) the constituent parts of behavioral characteristics through examining representative model parameters, and (*ii*) by quantifying the overall behavioral dissimilarity. Distance measures between behavior patterns *w*, and between experimental conditions *EC*, can be defined as model distances. One possible measure between models *λ*_*w*1 _and *λ*_*w*2_, for a set of observations **O**_*w*1 _and **O**_*w*2 _can be defined as

D(w1,w2)=12[L(w1,w2)+L(w2,w1)]
 MathType@MTEF@5@5@+=feaafiart1ev1aaatCvAUfKttLearuWrP9MDH5MBPbIqV92AaeXatLxBI9gBaebbnrfifHhDYfgasaacH8akY=wiFfYdH8Gipec8Eeeu0xXdbba9frFj0=OqFfea0dXdd9vqai=hGuQ8kuc9pgc9s8qqaq=dirpe0xb9q8qiLsFr0=vr0=vr0dc8meaabaqaciaacaGaaeqabaqabeGadaaakeaacqWGebarcqGGOaakcqWG3bWDdaWgaaWcbaGaeGymaedabeaakiabcYcaSiabdEha3naaBaaaleaacqaIYaGmaeqaaOGaeiykaKIaeyypa0ZaaSaaaeaacqaIXaqmaeaacqaIYaGmaaGaei4waSLaemitaWKaeiikaGIaem4DaC3aaSbaaSqaaiabigdaXaqabaGccqGGSaalcqWG3bWDdaWgaaWcbaGaeGOmaidabeaakiabcMcaPiabgUcaRiabdYeamjabcIcaOiabdEha3naaBaaaleaacqaIYaGmaeqaaOGaeiilaWIaem4DaC3aaSbaaSqaaiabigdaXaqabaGccqGGPaqkcqGGDbqxaaa@4DC3@

where *L*(*w*_1_, *w*_2_) is given by

L(w1,w2)=1T[log⁡P(Ow1|λw2)−log⁡P(Ow1|λw1)].
 MathType@MTEF@5@5@+=feaafiart1ev1aaatCvAUfKttLearuWrP9MDH5MBPbIqV92AaeXatLxBI9gBaebbnrfifHhDYfgasaacH8akY=wiFfYdH8Gipec8Eeeu0xXdbba9frFj0=OqFfea0dXdd9vqai=hGuQ8kuc9pgc9s8qqaq=dirpe0xb9q8qiLsFr0=vr0=vr0dc8meaabaqaciaacaGaaeqabaqabeGadaaakeaacqWGmbatcqGGOaakcqWG3bWDdaWgaaWcbaGaeGymaedabeaakiabcYcaSiabdEha3naaBaaaleaacqaIYaGmaeqaaOGaeiykaKIaeyypa0ZaaSaaaeaacqaIXaqmaeaacqWGubavaaGaei4waSLagiiBaWMaei4Ba8Maei4zaCMaemiuaaLaeiikaGccbeGae83ta80aaSbaaSqaaiabdEha3naaBaaameaacqaIXaqmaeqaaaWcbeaakiabcYha8HGaciab+T7aSnaaBaaaleaacqWG3bWDdaWgaaadbaGaeGOmaidabeaaaSqabaGccqGGPaqkcqGHsislcyGGSbaBcqGGVbWBcqGGNbWzcqWGqbaucqGGOaakcqWFpbWtdaWgaaWcbaGaem4DaC3aaSbaaWqaaiabigdaXaqabaaaleqaaOGaeiiFaWNae43UdW2aaSbaaSqaaiabdEha3naaBaaameaacqaIXaqmaeqaaaWcbeaakiabcMcaPiabc2faDjabc6caUaaa@5F23@

By quantifying behavioral comparisons between regulatory agents, studying combined effects of multiple regulatory agents may be guided with enhanced predictions. We envision a repository of MT dynamics data that can be probabilistically queried for behavioral similarities for a new regulatory agent, an isotype, or a combination. This can be done by evaluating *p*(**O**|*EC*) for an experimental condition *EC*, or evaluating *p*(**O**|*w*) for behavioral pattern *w*. Assuming that the tracking and modeling tasks were undertaken, a MT image database would contain a collection of individual MT tracks and model parameters representing *w*, in addition to original image sequences. Model based content retrieval provides additional advantages in query design. Hypothesized behaviors can be created and queried by manually selecting model parameters. Alternatively, query models can be estimated from a subset of MT tracks in the database.

**Figure 8:**
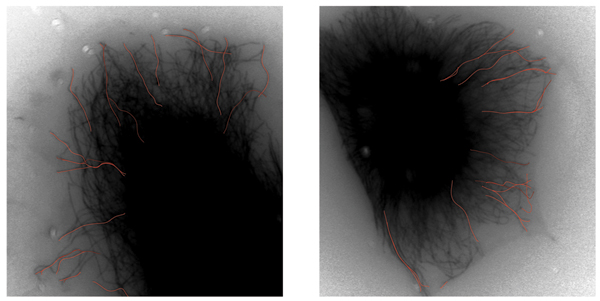
Tracked MTs superimposed on selected cells from *EC*_5_. Tracks were evaluated for their behavioral association to models representing *EC*_5 _by calculating *p*(*track*|*EC*_5_). Resulting probabilities were quantized to four categories to aid visibility. Darker tracks exhibit lower association with *EC*_5_, while brighter tracks are indicative of typical behaviors captured by models.

To study spatial relationships between MTs behaviorally, tracks can be grouped and visualized based on their behavior characteristics. For example, (Fig. 8) shows frames from *EC*_5_, with overlaid tracks. All tracks were evaluated for their similarity to conditions *EC*_4 _and *EC*_5_. In (Fig. 8), values of *p*(*track*|*EC*_5_) were quantized into four categories, indicated by four different shades of red channel, and were superimposed on MTs for illustration purposes. Darker shades indicate lower probability, e.g. behavioral association between the condition and the track.

This analysis provides the researcher with visual cues about regional dynamics within a cell. This may be especially important in studies of polarized cell types, such as neurons, where specific regional regulation of dynamics is critical to processes such as outgrowth and transport. Behavioral comparisons in adjacent populations may provide insight to the inner workings of flux between the soluble and polymeric tubulin fractions within the cytoplasm. The ratio between these two functionally distinct, but co-dependent phases may indicate cell-autonomous or drug-influenced regulation.

## Conclusion

MT dynamics research seeks to understand the complex mechanisms that underlie cytoskeletal responses to changes in environmental conditions. A clear understanding of the regulation of MT dynamic behavior may elucidate causal factors in various diseases and may reveal new therapeutic targets and strategies. In this work, we introduce novel data collection and analysis capabilities based on computer vision and machine learning tools. With the proposed methods, researchers can study MT dynamics with improved spatial and temporal quantification.

The most notable contribution of the proposed method is the novel analysis capabilities that are beyond the current state-of-the-art. Other contributions are the improvements over the manual data collection methods, such as higher accuracy (length along the MT vs Euclidean estimate), increased number of analyzable MT tracks, and objective consideration of all MT tracks at a fraction of the normally required time. Our preliminary results support manually established findings, and show that automated analysis of spatial and temporal patterns offers previously unattainable insights. Most notably, the standardization of data collection and analysis facilitates a comparative platform for future biological research.

As the volume and number of dynamics datasets has increased in recent years, similarities between the behavioral influence of MAPs and MTDs upon dynamics have emerged, leading to speculation of similar mechanisms. Dynamics models may facilitate the union of previously isolated MAP and MTD datasets, furthering our understanding of regulatory mechanisms of MTs.

Despite the difficulties inherent in fluorescence imaging, the proposed approach confirms manual findings in both track computation and in analysis. For example, due to photobleaching, observation durations were generally limited to only a few minutes with very low signal-to-noise ratios in images. With emerging techniques in microscopy and probes, such as the tip-binding proteins (EB1), much longer acquisition times will be possible with superior image quality. Our goal is to track all MTs in live cell images at longer durations. In this direction, the tracking method can be improved by reliably identifying all MTs individually. The nature of live cell MT images requires that frequent intersections, abrupt intensity variations on a single MT body, and focusing issues must be addressed adequately.

## Methods

The proposed analysis system evaluates MT dynamic behavior as a function of entire MT life histories through estimating statistical models from observations. A number of MT tracks per experimental condition is necessary for reliable estimates of model parameters. Thus, an automated tracking procedure was used in data collection.

### Automated tracking

**Figure 9:**
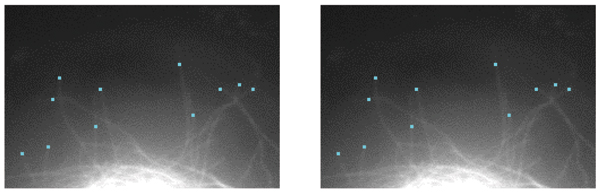
Example tip detection results in consecutive MT frames. Tip detection algorithm is sensitive to the proximity of the neighboring MTs. For example, tips that are close to MT intersections are eliminated due to uncertainty.

To achieve reliable models of MT behavior, numerous observations (MT tracks) are needed. Automated MT tracking provides a significant increase in analyzable data volume. The MT tracking problem has a short history in the literature, since live cell MT imaging has only been a mainstream research tool for about a decade. However, similar problems, such as the tracing of curvilinear structures in images, were previously addressed on neurons, blood vessels, roads, and so on. The most notable difference in MT images is the use of fluorescence, which presents additional difficulties in image analysis. For example, photobleaching, the gradual decay of fluorescence, causes illumination variations. Another issue is the additive nature of fluorescence. Overlapping MTs result in brighter regions in images, causing frequent over saturation. In (Fig. 9), such saturation is visible in lower regions of frames. Additionally, sample fluorescence exacerbates off-focus blur, which produces great challenges in detecting MT tips moving in and out of focus.

Previous work on automated MT detection and tracking include [16-18]. In [13,19], we described our tracking approach for live cell images and introduced the idea of model based analysis. In [16], the authors extract MT plus ends using a MT body and a tip model in a multi-scale operation. In [17] and [18], MTs are traced in segments from initially selected points and subsequently tracked. In [17], MTs are searched in a constrained space for tracking in subsequent frames.

**Figure 10:**
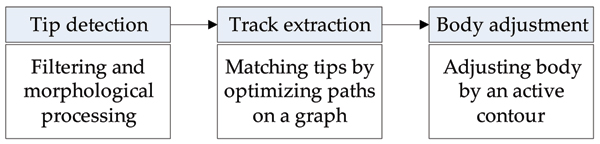
Conceptual overview of MT tracking procedure.

In this work, we used the tracking method from [13]. Conceptually, the proposed approach consists of three components, (Fig. 10). First, MT tip candidates are extracted in every frame of the image sequence. Then, tip correspondences between frames are established into MT tip tracks. Finally, the MT bodies are traced from the tips to extract dynamics information.

An automated MT tracking method should address the following: (*i*) highly variable tubule shapes, (*ii*) accurate estimation of the MT length considering the nonlinear shape, (*iii*) frequent occlusions and intersections from surrounding MTs, and (*iv*) low signal-to-noise ratios with spatial and temporal variations in illumination.

To address these issues, we consider MTs as flexible open curves in the image plane, with a fixed minus end near the nucleus and a dynamic plus end. Formally, a single MT is modeled by the open curve *C*(*s*), where *s *∈ [0, 1] is the curve parameter. The goal of the MT tracking task is to estimate the MT length by locating the tip and tracing the deformation of the MT body, in every frame.

#### Estimating microtubule tip positions

To address noise and illumination variations, we process the MT images with a line filter. Let *I *denote the intensity function in a frame, then the filter output is given by

If(x,y)=max⁡θ(I(x,y)∗G″σ,θ(x,y))
 MathType@MTEF@5@5@+=feaafiart1ev1aaatCvAUfKttLearuWrP9MDH5MBPbIqV92AaeXatLxBI9gBaebbnrfifHhDYfgasaacH8akY=wiFfYdH8Gipec8Eeeu0xXdbba9frFj0=OqFfea0dXdd9vqai=hGuQ8kuc9pgc9s8qqaq=dirpe0xb9q8qiLsFr0=vr0=vr0dc8meaabaqaciaacaGaaeqabaqabeGadaaakeaacqWGjbqsdaahaaWcbeqaaiabdAgaMbaakiabcIcaOiabdIha4jabcYcaSiabdMha5jabcMcaPiabg2da9maaxababaGagiyBa0MaeiyyaeMaeiiEaGhaleaaiiGacqWF4oqCaeqaaOGaeiikaGIaemysaKKaeiikaGIaemiEaGNaeiilaWIaemyEaKNaeiykaKIaey4fIOIafm4raCKbayaadaWgaaWcbaGae83WdmNaeiilaWIae8hUdehabeaakiabcIcaOiabdIha4jabcYcaSiabdMha5jabcMcaPiabcMcaPaaa@5076@

where the derivative of the Gaussian is taken along orientations *θ *at position (*x*, *y*), and *σ *is chosen as the average MT width. The maximum filter response, *I*^*f*^(*x*, *y*), is then binarized to generate a mask showing MT polymer mass. The binary mask is used for determining tip candidates in each frame. Example tip detection results from consecutive frames are shown in (Fig. 9).

Once the tip candidates are located in each frame, correspondences are established between frames by using a multi-frame graph matching algorithm. The reasoning behind formulating the correspondence as a graph optimization problem is that by matching multiple tips at once, occasional spurious tips are removed. Furthermore, the graph matching algorithm provides the flexibility of skipping frames, which handles missing tips between frames.

#### Extracting microtubule tip tracks

**Figure 11:**
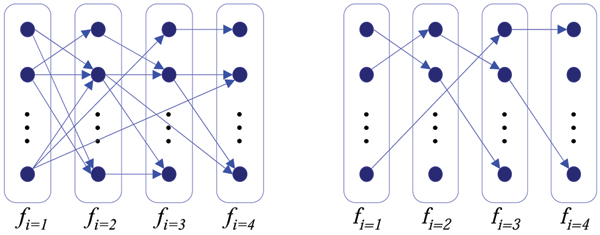
Example diagram of constructed graph, *G *= (*V*, *E*), across frames, *f*_*i*_, is shown in (a). A sample solution is shown in (b), where each path corresponds to a MT track.

Consider a MT time-lapse image sequence with *T *frames. Let *N*_*i *_denote the number of tip candidates detected in frame *i *for 1 ≤ *i *≤ *T*. Then, detected tips over the entire sequence can be individually denoted by tih
 MathType@MTEF@5@5@+=feaafiart1ev1aaatCvAUfKttLearuWrP9MDH5MBPbIqV92AaeXatLxBI9gBaebbnrfifHhDYfgasaacH8akY=wiFfYdH8Gipec8Eeeu0xXdbba9frFj0=OqFfea0dXdd9vqai=hGuQ8kuc9pgc9s8qqaq=dirpe0xb9q8qiLsFr0=vr0=vr0dc8meaabaqaciaacaGaaeqabaqabeGadaaakeaacqWG0baDdaqhaaWcbaGaemyAaKgabaGaemiAaGgaaaaa@30FE@ where *h *denotes the tip number in frame *i*, within the range 1 ≤ *h *≤ *N*_*i*_. We construct a graph *G *= (*V*, *E*) whose vertices *V *are the detected tip positions in frames 1..*T*, and the edges *E *represent the similarity of tip positions between frames. Thus, we represent tracks of MT tips with paths over *G*, (Fig. 11). Edges between vertices in non-consecutive frames are allowed, representing tracks with occasional missing tips.

To compute the similarity between tip positions in different frames, edge weights on *G*, we use the distance between tip positions constrained on a MT body. Note that the Euclidean distance cannot be used since different tips tend to move within close proximity of each other. Consider two tips tih
 MathType@MTEF@5@5@+=feaafiart1ev1aaatCvAUfKttLearuWrP9MDH5MBPbIqV92AaeXatLxBI9gBaebbnrfifHhDYfgasaacH8akY=wiFfYdH8Gipec8Eeeu0xXdbba9frFj0=OqFfea0dXdd9vqai=hGuQ8kuc9pgc9s8qqaq=dirpe0xb9q8qiLsFr0=vr0=vr0dc8meaabaqaciaacaGaaeqabaqabeGadaaakeaacqWG0baDdaqhaaWcbaGaemyAaKgabaGaemiAaGgaaaaa@30FE@ and tjr
 MathType@MTEF@5@5@+=feaafiart1ev1aaatCvAUfKttLearuWrP9MDH5MBPbIqV92AaeXatLxBI9gBaebbnrfifHhDYfgasaacH8akY=wiFfYdH8Gipec8Eeeu0xXdbba9frFj0=OqFfea0dXdd9vqai=hGuQ8kuc9pgc9s8qqaq=dirpe0xb9q8qiLsFr0=vr0=vr0dc8meaabaqaciaacaGaaeqabaqabeGadaaakeaacqWG0baDdaqhaaWcbaGaemOAaOgabaGaemOCaihaaaaa@3114@ in two different frames *f*_*i *_and *f*_*j*_. The main idea is to check if tih
 MathType@MTEF@5@5@+=feaafiart1ev1aaatCvAUfKttLearuWrP9MDH5MBPbIqV92AaeXatLxBI9gBaebbnrfifHhDYfgasaacH8akY=wiFfYdH8Gipec8Eeeu0xXdbba9frFj0=OqFfea0dXdd9vqai=hGuQ8kuc9pgc9s8qqaq=dirpe0xb9q8qiLsFr0=vr0=vr0dc8meaabaqaciaacaGaaeqabaqabeGadaaakeaacqWG0baDdaqhaaWcbaGaemyAaKgabaGaemiAaGgaaaaa@30FE@ and tjr
 MathType@MTEF@5@5@+=feaafiart1ev1aaatCvAUfKttLearuWrP9MDH5MBPbIqV92AaeXatLxBI9gBaebbnrfifHhDYfgasaacH8akY=wiFfYdH8Gipec8Eeeu0xXdbba9frFj0=OqFfea0dXdd9vqai=hGuQ8kuc9pgc9s8qqaq=dirpe0xb9q8qiLsFr0=vr0=vr0dc8meaabaqaciaacaGaaeqabaqabeGadaaakeaacqWG0baDdaqhaaWcbaGaemOAaOgabaGaemOCaihaaaaa@3114@ share a MT body between *f*_*i *_and *f*_*j*_. If tih
 MathType@MTEF@5@5@+=feaafiart1ev1aaatCvAUfKttLearuWrP9MDH5MBPbIqV92AaeXatLxBI9gBaebbnrfifHhDYfgasaacH8akY=wiFfYdH8Gipec8Eeeu0xXdbba9frFj0=OqFfea0dXdd9vqai=hGuQ8kuc9pgc9s8qqaq=dirpe0xb9q8qiLsFr0=vr0=vr0dc8meaabaqaciaacaGaaeqabaqabeGadaaakeaacqWG0baDdaqhaaWcbaGaemyAaKgabaGaemiAaGgaaaaa@30FE@ and tjr
 MathType@MTEF@5@5@+=feaafiart1ev1aaatCvAUfKttLearuWrP9MDH5MBPbIqV92AaeXatLxBI9gBaebbnrfifHhDYfgasaacH8akY=wiFfYdH8Gipec8Eeeu0xXdbba9frFj0=OqFfea0dXdd9vqai=hGuQ8kuc9pgc9s8qqaq=dirpe0xb9q8qiLsFr0=vr0=vr0dc8meaabaqaciaacaGaaeqabaqabeGadaaakeaacqWG0baDdaqhaaWcbaGaemOAaOgabaGaemOCaihaaaaa@3114@ do not belong to the same MT, then their similarity is insignificant. If tih
 MathType@MTEF@5@5@+=feaafiart1ev1aaatCvAUfKttLearuWrP9MDH5MBPbIqV92AaeXatLxBI9gBaebbnrfifHhDYfgasaacH8akY=wiFfYdH8Gipec8Eeeu0xXdbba9frFj0=OqFfea0dXdd9vqai=hGuQ8kuc9pgc9s8qqaq=dirpe0xb9q8qiLsFr0=vr0=vr0dc8meaabaqaciaacaGaaeqabaqabeGadaaakeaacqWG0baDdaqhaaWcbaGaemyAaKgabaGaemiAaGgaaaaa@30FE@ and tjr
 MathType@MTEF@5@5@+=feaafiart1ev1aaatCvAUfKttLearuWrP9MDH5MBPbIqV92AaeXatLxBI9gBaebbnrfifHhDYfgasaacH8akY=wiFfYdH8Gipec8Eeeu0xXdbba9frFj0=OqFfea0dXdd9vqai=hGuQ8kuc9pgc9s8qqaq=dirpe0xb9q8qiLsFr0=vr0=vr0dc8meaabaqaciaacaGaaeqabaqabeGadaaakeaacqWG0baDdaqhaaWcbaGaemOAaOgabaGaemOCaihaaaaa@3114@ belong to the same MT, then both growing and shortening cases should be considered between *f*_*i *_and *f*_*j*_. In the case of a growing MT, we project the position of tih
 MathType@MTEF@5@5@+=feaafiart1ev1aaatCvAUfKttLearuWrP9MDH5MBPbIqV92AaeXatLxBI9gBaebbnrfifHhDYfgasaacH8akY=wiFfYdH8Gipec8Eeeu0xXdbba9frFj0=OqFfea0dXdd9vqai=hGuQ8kuc9pgc9s8qqaq=dirpe0xb9q8qiLsFr0=vr0=vr0dc8meaabaqaciaacaGaaeqabaqabeGadaaakeaacqWG0baDdaqhaaWcbaGaemyAaKgabaGaemiAaGgaaaaa@30FE@ on *f*_*i *_to the same position on *f*_*j *_and compute the distance, *d*_*g*_(tih
 MathType@MTEF@5@5@+=feaafiart1ev1aaatCvAUfKttLearuWrP9MDH5MBPbIqV92AaeXatLxBI9gBaebbnrfifHhDYfgasaacH8akY=wiFfYdH8Gipec8Eeeu0xXdbba9frFj0=OqFfea0dXdd9vqai=hGuQ8kuc9pgc9s8qqaq=dirpe0xb9q8qiLsFr0=vr0=vr0dc8meaabaqaciaacaGaaeqabaqabeGadaaakeaacqWG0baDdaqhaaWcbaGaemyAaKgabaGaemiAaGgaaaaa@30FE@, tjr
 MathType@MTEF@5@5@+=feaafiart1ev1aaatCvAUfKttLearuWrP9MDH5MBPbIqV92AaeXatLxBI9gBaebbnrfifHhDYfgasaacH8akY=wiFfYdH8Gipec8Eeeu0xXdbba9frFj0=OqFfea0dXdd9vqai=hGuQ8kuc9pgc9s8qqaq=dirpe0xb9q8qiLsFr0=vr0=vr0dc8meaabaqaciaacaGaaeqabaqabeGadaaakeaacqWG0baDdaqhaaWcbaGaemOAaOgabaGaemOCaihaaaaa@3114@). We compute the shortening case, *d*_*s*_(tih
 MathType@MTEF@5@5@+=feaafiart1ev1aaatCvAUfKttLearuWrP9MDH5MBPbIqV92AaeXatLxBI9gBaebbnrfifHhDYfgasaacH8akY=wiFfYdH8Gipec8Eeeu0xXdbba9frFj0=OqFfea0dXdd9vqai=hGuQ8kuc9pgc9s8qqaq=dirpe0xb9q8qiLsFr0=vr0=vr0dc8meaabaqaciaacaGaaeqabaqabeGadaaakeaacqWG0baDdaqhaaWcbaGaemyAaKgabaGaemiAaGgaaaaa@30FE@, tjr
 MathType@MTEF@5@5@+=feaafiart1ev1aaatCvAUfKttLearuWrP9MDH5MBPbIqV92AaeXatLxBI9gBaebbnrfifHhDYfgasaacH8akY=wiFfYdH8Gipec8Eeeu0xXdbba9frFj0=OqFfea0dXdd9vqai=hGuQ8kuc9pgc9s8qqaq=dirpe0xb9q8qiLsFr0=vr0=vr0dc8meaabaqaciaacaGaaeqabaqabeGadaaakeaacqWG0baDdaqhaaWcbaGaemOAaOgabaGaemOCaihaaaaa@3114@), in the same way. Then, the weight on *G *between vertices tih
 MathType@MTEF@5@5@+=feaafiart1ev1aaatCvAUfKttLearuWrP9MDH5MBPbIqV92AaeXatLxBI9gBaebbnrfifHhDYfgasaacH8akY=wiFfYdH8Gipec8Eeeu0xXdbba9frFj0=OqFfea0dXdd9vqai=hGuQ8kuc9pgc9s8qqaq=dirpe0xb9q8qiLsFr0=vr0=vr0dc8meaabaqaciaacaGaaeqabaqabeGadaaakeaacqWG0baDdaqhaaWcbaGaemyAaKgabaGaemiAaGgaaaaa@30FE@ and tjr
 MathType@MTEF@5@5@+=feaafiart1ev1aaatCvAUfKttLearuWrP9MDH5MBPbIqV92AaeXatLxBI9gBaebbnrfifHhDYfgasaacH8akY=wiFfYdH8Gipec8Eeeu0xXdbba9frFj0=OqFfea0dXdd9vqai=hGuQ8kuc9pgc9s8qqaq=dirpe0xb9q8qiLsFr0=vr0=vr0dc8meaabaqaciaacaGaaeqabaqabeGadaaakeaacqWG0baDdaqhaaWcbaGaemOAaOgabaGaemOCaihaaaaa@3114@ is computed as

Sim(tih,tjr)=e−min⁡(dg,ds).
 MathType@MTEF@5@5@+=feaafiart1ev1aaatCvAUfKttLearuWrP9MDH5MBPbIqV92AaeXatLxBI9gBaebbnrfifHhDYfgasaacH8akY=wiFfYdH8Gipec8Eeeu0xXdbba9frFj0=OqFfea0dXdd9vqai=hGuQ8kuc9pgc9s8qqaq=dirpe0xb9q8qiLsFr0=vr0=vr0dc8meaabaqaciaacaGaaeqabaqabeGadaaakeaacqWGtbWucqWGPbqAcqWGTbqBcqGGOaakcqWG0baDdaqhaaWcbaGaemyAaKgabaGaemiAaGgaaOGaeiilaWIaemiDaq3aa0baaSqaaiabdQgaQbqaaiabdkhaYbaakiabcMcaPiabg2da9iabdwgaLnaaCaaaleqabaGaeyOeI0IagiyBa0MaeiyAaKMaeiOBa4MaeiikaGIaemizaq2aaSbaaWqaaiabdEgaNbqabaWccqGGSaalcqWGKbazdaWgaaadbaGaem4CamhabeaaliabcMcaPaaakiabc6caUaaa@4CE6@

Once *G *is constructed, we compute a maximum weight matching of *G *where paths correspond to MT tracks. In graph theory, a *vertex disjoint path cover C *is a covering of *G *where each vertex of *G *is in one and only one path of *C*. The weight of a path cover is defined as the sum of weights on its edges. Using the notion of path cover, the problem of finding the best MT tracks corresponds to finding the *maximum weight path cover *of *G *with the weights defined by the similarity in (4). Formally, a maximum weight path cover *C*(*G*) is a path cover which satisfies

C(G)=arg⁡max⁡CiW(Ci)
 MathType@MTEF@5@5@+=feaafiart1ev1aaatCvAUfKttLearuWrP9MDH5MBPbIqV92AaeXatLxBI9gBaebbnrfifHhDYfgasaacH8akY=wiFfYdH8Gipec8Eeeu0xXdbba9frFj0=OqFfea0dXdd9vqai=hGuQ8kuc9pgc9s8qqaq=dirpe0xb9q8qiLsFr0=vr0=vr0dc8meaabaqaciaacaGaaeqabaqabeGadaaakeaacqWGdbWqcqGGOaakcqWGhbWrcqGGPaqkcqGH9aqpcyGGHbqycqGGYbGCcqGGNbWzdaWfqaqaaiGbc2gaTjabcggaHjabcIha4bWcbaGaem4qam0aaSbaaWqaaiabdMgaPbqabaaaleqaaOGaem4vaCLaeiikaGIaem4qam0aaSbaaSqaaiabdMgaPbqabaGccqGGPaqkaaa@422C@

where W(Ci)=∑euv∈CiSim(euv)
 MathType@MTEF@5@5@+=feaafiart1ev1aaatCvAUfKttLearuWrP9MDH5MBPbIqV92AaeXatLxBI9gBaebbnrfifHhDYfgasaacH8akY=wiFfYdH8Gipec8Eeeu0xXdbba9frFj0=OqFfea0dXdd9vqai=hGuQ8kuc9pgc9s8qqaq=dirpe0xb9q8qiLsFr0=vr0=vr0dc8meaabaqaciaacaGaaeqabaqabeGadaaakeaacqWGxbWvcqGGOaakcqWGdbWqcqWGPbqAcqGGPaqkcqGH9aqpdaaeqaqaaiabdofatjabdMgaPjabd2gaTjabcIcaOiabdwgaLnaaBaaaleaacqWG1bqDcqWG2bGDaeqaaOGaeiykaKcaleaacqWGLbqzdaWgaaadbaGaemyDauNaemODayhabeaaliabgIGiolabdoeadnaaBaaameaacqWGPbqAaeqaaaWcbeqdcqGHris5aaaa@4791@ and *u*, *v *are two vertices in *G *for which the similarity is computed as in Eq.(4). Note that between two frames the best tracks can be computed as the maximum match of a bipartite graph. However, for multiple frames, the problem becomes NP-hard. Here, we adopt the approximation proposed in [20].

The described method is sufficient to track MT tips between different frames. However, without tracing the MT body, the best estimates of MT growth and shortening would be limited to Euclidean approximations between tip positions, (see Current analysis method). Since in live cell images, the MT body is typically non-linear, this approximation is a rough one in practice. Instead, we determine the MT body length in all frames.

#### Estimating microtubule body

In essence, we compute the MT body length along the body in each frame and determine the growth and shortening as consecutive length differences. Given the tip positions in each frame, we estimate the deformable curve constituting the MT body between these tips and a fixed point along the MT body. Note that the fixed point does not have to lie on the body of a specific MT for the purposes of computing the growth and shortening. In cases where the fixed point lies on another MT rather than the MT being measured, the resulting change in length is still a better estimate than the Euclidean case, so long as the fixed point taken consistently across frames. Details of fixing this point can be found in [13]. Due to the constant deformations, the fixed point location may exhibit small variations, (Fig. 5d – 5f). This is the major contributor of errors in length estimation between frames. Finally, based on the estimated plus and minus ends of the MT, the MT body is extracted using active contours with ridge features.

### Model based analysis

A number of studies examined physical models for MT structure and dynamics. We refer the interested reader to [21-23], and the references therein, for a review of previous models of MT dynamic instability. For example, in [23], the authors use a simulation model to investigate the fluctuations in tubulin concentration in relation to MT dynamics. In contrast to previous dynamics models, we propose using machine learning methods for modeling various *MT behavior patterns *occurring in different experimental conditions.

**Figure 12:**
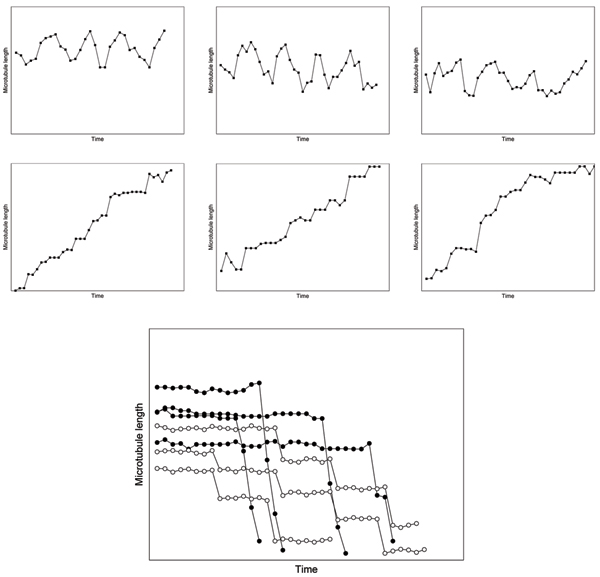
Example life history plots from hypothetical MTs showing different behaviors. Life histories were inspired by [33]. Individual MTs in the top undergo several length excursions, while the MTs in the middle row exhibit an overall growth tendency. The bottom chart shows individual MTs, distinguished by filled and open circles, which are superimposed on the time axis for visual comparison. While both groups of MTs display shortening, the group indicated by the open circles shorten gradually as compared with the rest of the MTs.

MT behavior can be considered as a random process that evolves in time. For example, (Fig. 12) shows different behaviors of hypothetical MTs from different MT populations. MTs in the middle row exhibit a growth tendency, while MTs in the top row show several length excursions within the same amount of time. The bottom chart shows two different shortening MT groups for visual comparison of behavior patterns.

Automated tracking is sufficient to quantify traditional dynamics parameters. We propose an analysis approach targeting behavioral information beyond what is provided by the traditional parameters. We begin with including contextual information in time. In other words, as opposed to parsing the growth and shortening events out of MT tracks (life histories), we keep the MT tracks intact. Therefore, each MT track is treated as an observation from some *behavior pattern*. For example, the tracks in (Fig. 12, top row and middle row) are observation instances from different behavior patterns. Thus, if *g *denotes a small, and *G *denotes a large growth events, then the observed tracks, *ggggGGGG *and *ggGGggGG *should be treated as different behaviors even if the average growth rates may be equal. This definition of a MT *behavior pattern *leads to new analysis capabilities. Each behavior pattern can be described by a model. Subsequently, estimated models are used in analyzing MT dynamic behavior; for instance, in evaluating dynamic similarities between MT populations.

In modeling the MT dynamic behavior, biological insights provide essential guidance. Similar behavior patterns are known to be shared between different experimental conditions, while MT populations within a cell may exhibit dissimilar patterns. Thus, modeling design should handle expected variations of behavior within each experimental condition, and similarities between different experimental conditions.

Formally, we denote each experimental condition by *EC*, consisting of groups of behavior patterns, *w*. All experimental conditions have a known label, while patterns making up a condition are unknown. The problem is to estimate a model *λ *for each pattern *w*, such that differences between *EC*_*i *_and *EC*_*j*_, *i *≠ *j*, are emphasized, while each pattern may occur in different experimental conditions, *w *∈ *EC*_*i *_and *w *∈ *EC*_*j*_. Note that our formulation calls for a discriminative approach between *EC*, while descriptive models of *w *is the goal across different *EC*'s.

A well known class of models used in representing activity is the Hidden Markov Models (HMMs). In the past, they have been used in numerous applications, most notably in speech recognition, [24], and in genomic sequence analysis, [25-28]. Particularly in activity context, HMMs were used in activity recognition [29], abnormal activity detection, gesture recognition, and American Sign Language recognition. In the next section we review the essentials of HMMs, while referring the reader to [24] for further details.

### Hidden Markov models

HMMs are probabilistic generative models estimating the statistics of a process from observation sequences generated by that process. The modeled process is assumed to be not directly observable, thus hidden states capture statistics of the process, subject to stochastic constraints. In practice, hidden states generally correspond to certain physical characteristics of the process. Detailed information on modeling with HMMs can be found in [24,28]. Concisely, HMMs, denoted by *λ*, are described by parameters *λ *= (*π*, *A*, *B*), where *π *is the state priors, *A *is the transition, and *B *is the emission probabilities. Given an observation sequence *O *= (*o*_1_, *o*_2_,..., *o*_*T*_), where *t *= 1..*T *denotes time, and a model *λ *= (*π*, *A*, *B*), the quantity *P*(*O*|*λ*) can be computed efficiently. Given a set of observation sequences, estimating the parameters of *λ *is generally performed using maximum likelihood methods, while discriminative techniques were suggested in classification tasks, [30,31].

### Modeling microtubule dynamics by HMMs

From the biological perspective, classification of tracks to respective *EC *is not the end goal for dynamics analysis since labels of *EC *are known a priori. However, estimated behavior models, *λ*, provide novel analytical capabilities. Furthermore, model parameters may reveal further insights into MT dynamic behavior. Our formulation of the problem aims to extract behavior patterns through estimating *λ*, while discriminating between different *EC*. In doing so, we employ the classification score as our measure of model reliability. The problem description motivates us to use a model based clustering approach to estimate a *λ *for each *w*. HMM based clustering methods are discussed in [32].

After parameter estimation, each *EC *is represented by a mixture of *λ *where dynamics variations within each *EC *are modeled by the components of the mixture. In this sense, each *λ *models the (pseudo-)center of a *w*, the component behavior patterns contributing to the resulting behavior in respective *EC*. The estimation of *λ *is primarily a modeling task, while discrimination between *w *is handled by clustering the observations, MT tracks, into behavior patterns represented by the respective *w*.

#### Model estimation

We define the quantity *P*(**O**|*λ*) as the similarity measure between the observation sequences *O *and the cluster center *λ*_*w *_of dynamics category *w*. Expected overall likelihood

L=∑w∑o∈Cwlog⁡P(O|λw)
 MathType@MTEF@5@5@+=feaafiart1ev1aaatCvAUfKttLearuWrP9MDH5MBPbIqV92AaeXatLxBI9gBaebbnrfifHhDYfgasaacH8akY=wiFfYdH8Gipec8Eeeu0xXdbba9frFj0=OqFfea0dXdd9vqai=hGuQ8kuc9pgc9s8qqaq=dirpe0xb9q8qiLsFr0=vr0=vr0dc8meaabaqaciaacaGaaeqabaqabeGadaaakeaacqWGmbatcqGH9aqpdaaeqbqaamaaqafabaGagiiBaWMaei4Ba8Maei4zaCMaemiuaaLaeiikaGccbeGae83ta8KaeiiFaWhcciGae43UdW2aaSbaaSqaaiabdEha3bqabaGccqGGPaqkaSqaaiabd+gaVjabgIGiolabdoeadnaaBaaameaacqWG3bWDaeqaaaWcbeqdcqGHris5aaWcbaGaem4DaChabeqdcqGHris5aaaa@4746@

is maximized through

• Repartition

- assign *o *to cluster *C*_*w *_such that

*w *= arg max_*w' *_log *p*(*o*|*λ*_*w'*_)

• Reestimate models

- train *λ*_*w *_on *C*_*w*_, *w *= 1..*W*

In each iteration of the algorithm, observation *o *is assigned to maximally likely cluster *C*_*w*_, whose center *λ*_*w *_is re-estimated using the new members of *C*_*w*_. The iterations are terminated when no significant increase in the overall likelihood is observed.

#### Model evaluation

As mentioned, we utilize the classification accuracy between *EC *as our measure for overall model reliability. We compute the probability *p*(*o*|*EC*) by

p(o|EC)=∑w=1Wp(o|λw,EC)Pw
 MathType@MTEF@5@5@+=feaafiart1ev1aaatCvAUfKttLearuWrP9MDH5MBPbIqV92AaeXatLxBI9gBaebbnrfifHhDYfgasaacH8akY=wiFfYdH8Gipec8Eeeu0xXdbba9frFj0=OqFfea0dXdd9vqai=hGuQ8kuc9pgc9s8qqaq=dirpe0xb9q8qiLsFr0=vr0=vr0dc8meaabaqaciaacaGaaeqabaqabeGadaaakeaacqWGWbaCcqGGOaakcqWGVbWBcqGG8baFcqWGfbqrcqWGdbWqcqGGPaqkcqGH9aqpdaaeWbqaaiabdchaWjabcIcaOiabd+gaVjabcYha8HGaciab=T7aSnaaBaaaleaacqWG3bWDcqGGSaalcqWGfbqrcqWGdbWqaeqaaOGaeiykaKIaemiuaa1aaSbaaSqaaiabdEha3bqabaaabaGaem4DaCNaeyypa0JaeGymaedabaGaem4vaCfaniabggHiLdaaaa@4BE9@

where *P*_*w *_is the relative number of cluster members, and estimate the separation by counting the number of correctly classified tracks *o *∈ **O **using the decision rule

EC∗=arg⁡max⁡i[P(o|ECi)].
 MathType@MTEF@5@5@+=feaafiart1ev1aaatCvAUfKttLearuWrP9MDH5MBPbIqV92AaeXatLxBI9gBaebbnrfifHhDYfgasaacH8akY=wiFfYdH8Gipec8Eeeu0xXdbba9frFj0=OqFfea0dXdd9vqai=hGuQ8kuc9pgc9s8qqaq=dirpe0xb9q8qiLsFr0=vr0=vr0dc8meaabaqaciaacaGaaeqabaqabeGadaaakeaacqWGfbqrcqWGdbWqdaahaaWcbeqaaiabgEHiQaaakiabg2da9iGbcggaHjabckhaYjabcEgaNnaaxababaGagiyBa0MaeiyyaeMaeiiEaGhaleaacqWGPbqAaeqaaOGaei4waSLaemiuaaLaeiikaGIaem4Ba8MaeiiFaWNaemyrauKaem4qam0aaSbaaSqaaiabdMgaPbqabaGccqGGPaqkcqGGDbqxcqGGUaGlaaa@47A5@

Note that the decision is conditional on *λ*_*w*_, *EC*, representing contributions of each member *λ*_*w *_of *EC*.

## Competing interests

The authors declare that they have no competing interests.

## Authors' contributions

AA carried out the quantification and modeling of dynamics. AJP and EK performed data collection and manual tracking of MTs. LW, SCF, BSM, KR participated in the design and coordination of the study, as well as critical reading of the manuscript.
